# Comparative Analyses of Fecal Microbiota in European Mouflon (*Ovis orientalis musimon*) and Blue Sheep (*Pseudois nayaur*) Living at Low or High Altitudes

**DOI:** 10.3389/fmicb.2019.01735

**Published:** 2019-07-30

**Authors:** Guolei Sun, Honghai Zhang, Qinguo Wei, Chao Zhao, Xiufeng Yang, Xiaoyang Wu, Tian Xia, Guangshuai Liu, Lei Zhang, Ying Gao, Weilai Sha, Ying Li

**Affiliations:** ^1^College of Life Science, Qufu Normal University, Qufu, China; ^2^Wild World Jinan, Jinan, China

**Keywords:** European mouflon, blue sheep, gut microbiota, 16S rRNA gene, altitude

## Abstract

The gut microbiota is a complex and essential system organ that plays an integrative role in balancing key vital functions in the host. Knowledge of the impact of altitude on the gut microbiota of European mouflon (*Ovis orientalis musimon*) and blue sheep (*Pseudois nayaur*) is currently limited. In this study, we compared the characteristics of gut microbiota in 5 mouflon at low altitude (K group), 4 mouflon at high altitude (L group), 4 blue sheep at low altitude (M group), and 4 blue sheep at high altitude (N group). The V3–V4 region of the 16S rRNA gene was analyzed using high-throughput sequencing. Analyses based on the operational taxonomic units showed significant changes in the gut microbial communities between groups at different altitudes. At the phylum level, groups at the high altitudes had a higher relative abundance of Firmicutes and a lower relative abundance of Bacteroidetes than those at the low altitudes. A higher Firmicutes:Bacteroidetes ratio is beneficial to animals in terms of the gut microbiota-mediated energy harvest. The relative abundance of Proteobacteria was significantly higher in the gut microbiota of mouflon sheep at high altitudes. At the genus level, the *Bacteroides:Prevotella* ratio was significantly higher in the low-altitude group (than the high-altitude group) of mouflon sheep and the ratio was significantly higher in the high-altitude group (than the low-altitude group) in blue sheep. In addition, the *Ruminococcaceae_UCG-005* related to cellulose and starch digestion was the predominant genus in blue sheep and the relative abundance of the genus was significant higher in the high-altitude group than the low-altitude group of blue sheep (*P* < 0.01). In conclusion, our results suggested that the gut microbiota of high-altitude groups of sheep had stronger abilities related to energy metabolism and the decomposition of substances, e.g., fiber and cellulose, and that such abilities are associated with high-altitude adaptation.

## Introduction

Natural environmental factors can have significant effects on all biological aspects of an animal. Animals are often able to adapt to different environments through physiological or behavioral changes. In many animals, a huge number of microbes reside in the gastrointestinal tract and form a complex gut microbiome. Hosts provide a living environment for the microbial communities of the gut, while gut microbiota contributes to nutrient absorption of the host. The gut microbiota plays an integrative role in balancing key vital functions in the host, such as metabolism, inflammation, and immune function (Round and Mazmanian, 2009; Sekirov et al., 2010; Qin et al., 2012; Tremaroli and Bäckhed, 2012). A number of studies have assessed the bacterial community structure and function of the gut microbiota in herbivores (Castillo et al., 2007; Bergmann et al., 2015; Miller et al., 2016; Avila-Jaime et al., 2018). Coevolutionary modes tend to occur when interactions develop between the gut microbiota and hosts (Maynard et al., 2012). Considering the importance of gut microbiota, the identification of factors that affect animal gut microbial flora may have broad implications in the fields of biology, ecology, veterinary science, and more.

The blue sheep (*Pseudois nayaur*) is a medium-sized herbivore and a species of Bovidae that includes two subspecies: *P. nayaur* and *P. szechuanensis* (Marschall, 1998). Blue sheep are distributed throughout Bhutan, China (Tibetan Plateau and the surrounding mountain regions), northern India and Myanmar, Nepal, and northern Pakistan (Shackleton, 1997; Rabinowitz and Khaing, 1998). In addition, blue sheep inhabit high mountainous areas [>4000 m above sea level (a.s.l.)] with woodland steppe or bare rocks as the main habitat types, that are far from water resources (Liu et al., 2018).

Although blue sheep are listed as a Least Concern (LC, Version 3.1) species by the International Union for Conservation of Nature (Harris, 2014) due to its wide distribution, the population structure of the species is still unclear due to the difficulty in conducting estimates in the steep habitats. The total population size of this species is roughly estimated to be 47000–414000 (Fox et al., 1991; Schaller, 1998; Wang, 1998; Liu et al., 2005). In China, the number of blue sheep in the Tibetan Plateau, the main habitat for blue sheep, were estimated and found to have been dramatically declining from 1991 to 2002 (Harris and Loggers, 2004). As a consequence, blue sheep were listed as a second-grade state protection animal and an Endangered class.

The European mouflon (*Ovis orientalis musimon*) is a subspecies of *O. orientalis*, a member of the Bovidae, and is considered to be the ancestor of the modern-day sheep (Valdez and Batten, 1982; Groves, 1993; Rezaei et al., 2010). Due to the drastic decline in the population as a result of hunting, environmental changes, and habitat deterioration, the European mouflon is listed as a Vulnerable species under criterion A2cde by the Valdez (2008). Notably, *O. orientalis* is listed on CITES Appendix I. The mouflon is a herbivorous animal that feeds on grasses and shrubs and inhabits moderate or very arid grasslands that generally occur below 2500 m a.s.l. (Hoefs, 1985).

Blue sheep and mouflon can adapt to complex and hostile environments. Although the two species have strong adaptive abilities to different conditions, their population size were still small. And previous studies have mainly concentrated on their taxonomy, population, distribution, habitats, and ecology (Yuqun, 1990; Hermans, 1996; Shackleton et al., 1997; Namgail et al., 2009; Tan et al., 2012). However, few studies have been conducted on their molecular biology to determine the taxonomic and biological mechanisms (Zeng et al., 2008; Tan et al., 2012; Barbato et al., 2017; Yang et al., 2019).

“Altitude” is an important factor that can affect and shape gut microbial communities. Specifically, altitude can affect the composition and structure of gut microbiota in mammals (Li K. et al., 2016; Karl et al., 2018; Zhao et al., 2018). Low barometric pressure, low temperature, and high radiation conditions are physiologic stressors that can affect both the survival and reproduction of organisms (Hochachka et al., 1996; Cheviron et al., 2012). In addition, intense evolutionary selection pressures can cause a variety of physiological changes in animals (Storz et al., 2010; Ivy and Scott, 2015; Simonson, 2015). Environmental change can also alter the gut microbiota, studies have shown that short exposure to high-altitude can change the composition of gut microbiota.

However, studies using high-throughput sequencing technology to assess the gut microbiota of Bovidae are relatively insufficient, and studies on the gut microbiota of blue sheep and mouflon sheep are greatly lacking. Therefore, we conducted a gut microbiota analysis using samples of blue sheep and mouflon sheep collected from low- (<100 m a.s.l.) and high- (>2300 m a.s.l.) altitude habitats. In this study, we aimed to identify the effects of altitudinal variation on the gut microbial flora of mouflon and blue sheep to provide greater insights into the gut microbiota community composition and structure of animals that normally inhabit different altitudes. And the research will be benefit to adaptive management and the evaluation of conservation efficacy of the two species.

## Materials and Methods

### Ethics Statement

This study was carried out and executed in accordance with the recommendations of the Guide to Animal Experiments of the Ministry of Science and Technology (Beijing, China). All experiments involving animals were approved by the Qufu Normal University Institutional Animal Care and Use Committee (Permit Number: QFNU2018-031).

### Sample Collection

Samples were collected from the Ji’nan Wild Zoo (Shandong Province, China) and Xining Wildlife Park (Qinghai Province, China), which are positioned at low altitudes (<100 m a.s.l.) and high altitudes (>2000 m a.s.l.), respectively. Detail information of the two study species and the groupings are listed in Table 1. The age of all study animals ranged from 3 to 8 years, and the subjects were healthy, free of diseases, and had not been on any medications for at least 3 months prior to the sampling. Both the species from the different locations were fed roughly the same diets, which were primarily comprised of cotton grass, alfalfa, carrots, and pellet feed. Fresh fecal samples were obtained in the early mornings after the barns were cleaned. Samples were collected within half an hour of the aseptic cleaning process. The fecal samples were frozen to cryogenic temperatures for immediate storage and then frozen to −80°C before further processing and analysis.

**TABLE 1:** Information for fecal samples from European mouflon and blue sheep.

**Species**	**Group**	**Sample name**	**Sex (M/F)**	**Place**
European mouflon	K	M1	M	Ji’nan Wild Zoo
		M2	F	
		M3	F	
		M4	F	
		M5	F	
	L	M6	F	Xining Wildlife Park
		M7	F	
		M8	M	
		M9	M	
Blue sheep	M	B1	F	Ji’nan Wild Zoo
		B2	F	
		B3	M	
		B4	M	
	N	B6	M	Xining Wildlife Park
		B7	M	
		B8	F	
		B9	F	

*K, low-altitude mouflon; L, high-altitude mouflon; M, low-altitude blue sheep; N, high-altitude blue sheep.*

### DNA Extraction

Genomic DNA extraction was performed using QIAamp^®^ Stool Mini Kit (Qiagen, Germany) according to manufacturer’s recommendations. A Nanodrop UV-Vis Spectrophotometer (Thermo Fisher scientific) was used to determine the amount of genomic DNA.

### 16S rRNA Gene PCR and Sequencing

To characterize the resident microbial community, we amplified and analyzed the 16S rRNA gene of the V3–V4 region with the forward primer (CCTAYGGGRBGCASCAG) and the reverse primer (GGACTACNNGGGTATCTAAT). The final volume of the amplification reaction mixture was 50 μl and contained 5 μl microbial genomic DNA, 5 μl of each primer, 25 μl KAPA HiFi HotStart Ready Mix (KAPA Biosystems), and 10 μl ddH_2_O. The following PCR conditions were used to acquire the products: 95°C for 10 min, followed by 25 cycles at 94°C for 30 s, annealing at 55°C for 30 s, elongation at 72°C for 30 s, and a final elongation step at 72°C for 5 min. The DNA fragments of the products were ∼410 bp in size and were analyzed using agarose gel electrophoresis to confirm the quantity and quality of the products. The PCR products were then purified using AMPure XP beads according to the manufacturer’s instructions. Before sequencing, we used TruSeq DNA PCR-free Sample Preparation Kit (Illumina, United States) to generate the sequencing library. Samples were sequenced using the Illumina HiSeq 2500 platform (Illumina) with the 250 bp. A pair-ended running mode.

### Sequence Processing and Analysis

Raw reads from the Illumina MiSeqPE250 were cleaned up and assembled in the following steps. Barcodes and primer sequences were cut off and raw tags were obtained and performed using FLASH (Magoč and Salzberg, 2011) and QIIME (Version 1.7.0) (Caporaso et al., 2010). The UCHIME algorithm (Edgar et al., 2011) was used to remove the chimeric sequences from the raw tags and effective tags were obtained. We assessed the community composition by classifying the sequences (Uparsev7.0.1001) into operational taxonomic units (OTUs) defined by whether the 16S rRNA sequence similarity was greater than or equal to 97% (Edgar, 2013). Finally, species annotations were performed using the GreenGene Database (DeSantis et al., 2006) with the RDP classifier algorithm (Version 2.2) (Wang et al., 2007).

The alpha-diversity of microbial communities was determined using different indices (Chao1, ACE, Shannon, Simpson, Coverage) and calculated with QIIME (V1.7.0). Furthermore, the bacterial community diversity was analyzed using rarefaction and rank-abundance plots, and displayed using R software. Tukey and Wilcoxon’s tests were used for statistical analysis. Differences among samples were assessed using a Principal Component Analysis (PCA), Principal Coordinates Analysis (PCoA), and non-metric multidimensional scaling (NMDS), and displayed using R software. Distance-based methods, such as the Unweighted Pair Group Method with Arithmetic Mean (UPGMA) have been used to conduct cluster analyses based on the similarity and dissimilarity of bacterial communities among samples. Linear discriminant analysis (LDA) effect size (LEfSe) analysis (Segata et al., 2011) was used to detect statistically significant differences in the species between the study groups.

Next-generation sequencing datasets have been deposited in the NCBI sequence read archive (SRA) with accession number: PRJNA511517.

## Results

### Overview of the Sequencing Data

**FIGURE 1:**
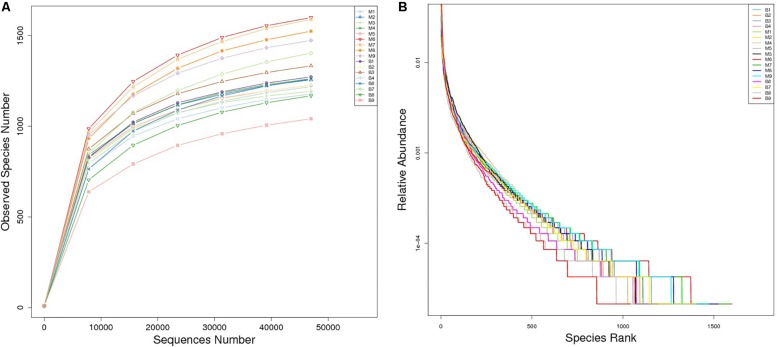
Rarefaction curves **(A)** and rank abundance curves **(B)** of each sample. The saturated rarefaction curves and species richness indices indicate that the sampling was comprehensive. In rank abundance curves, a wider span of curves reflects a higher relative species abundance, and the smoother the curve on the *y*-axis reveals a higher evenness of bacterial species in the fecal samples of European mouflon and blue sheep.

A total of 1,377,737 high quality reads were produced from 17 fecal samples and were classified into 2774 OTUs after conducting quality control with 97% similarity. The alpha diversity of the microbial communities was assessed and calculated (Table 2). Rarefaction curves (Figure 1A) of OTU richness were calculated using the vegan library in the R statistical computing language (Oksanen et al., 2013). When the rarefaction curves approached a plateau, this suggested that the number of OTUs was sufficient to reveal the authentic bacterial communities within each sample. Furthermore, ranked abundance curves (Figure 1B) were used to analyze the community diversity, which indicated both the evenness and abundance of species in the samples.

**TABLE 2:** Alpha-diversity of gut microbiota in fecal samples from European mouflon and blue sheep.

**Sample**	**Observed species**	**Shannon**	**Simpson**	**Chao1**	**ACE**	**Goods coverage**
M1	1178	7.697	0.983	1254.507	1267.801	0.997
M2	1256	8.218	0.992	1333.166	1351.258	0.997
M3	1193	8.360	0.994	1246.082	1257.676	0.997
M4	1262	8.416	0.993	1361.365	1373.270	0.996
M5	1226	8.258	0.992	1310.645	1317.484	0.997
M6	1599	7.984	0.981	1708.591	1730.618	0.995
M7	1589	8.160	0.987	1738.487	1773.205	0.994
M8	1524	8.275	0.991	1671.000	1677.746	0.995
M9	1473	8.350	0.992	1595.112	1594.821	0.996
B1	1272	8.212	0.992	1355.563	1366.844	0.997
B2	1226	8.008	0.989	1303.260	1328.637	0.997
B3	1333	8.279	0.992	1408.612	1443.001	0.996
B4	1218	8.019	0.989	1291.845	1322.900	0.997
B6	1260	7.679	0.986	1349.644	1375.531	0.996
B7	1403	8.004	0.988	1563.159	1597.229	0.995
B8	1168	7.181	0.976	1329.183	1302.100	0.996
B9	1041	7.240	0.980	1146.062	1177.697	0.996

### Bacteria Composition and Relative Abundance

A total of 33 phyla, 64 classes, 121orders, 209 families, 378 genera, and 108 species were detected in the prokaryotic microbiota communities from all fecal samples from both blue sheep and the mouflon.

At the phylum level, the top five ranked abundance-based phyla in the mouflon sheep were Firmicutes (57.25%), Bacteroidetes (33.66%), Proteobacteria (2.14%), Spirochaetes (1.63%), and Verrucomicrobia (1.57%) at low altitudes (group K); and Firmicutes (62.13%), Bacteroidetes (21.07%), Proteobacteria (9.21%), Fibrobacteres (2.90%), and Spirochaetes (1.37%) at high altitudes (group L).

The top five ranked abundance-based phyla in the mouflon were Firmicutes (57.31%), Bacteroidetes (33.48%), Proteobacteria (2.29%), Spirochaetes (2.14%), and Fibrobacteres (1.30%) at low altitudes (group M); and Firmicutes (61.09%), Bacteroidetes (28.30%), Proteobacteria (2.94%), Fibrobacteres (2.62%), and Spirochaetes (1.89%) at high altitudes (group N).

In summary, the most predominant phyla in all groups (K, L, M, and N) were Firmicutes, Bacteroidetes, and Proteobacteria; the proportion of which comprised more than 92% of the total composition of the samples. It is note worthy that Verrucomicrobia was a unique phylum that occurred in the top five ranked phyla in group K, while the others contained the phylum Fibrobacteres. The rank of the community structure and composition of the top five phyla was completely consistent within mouflon and blue sheep.

At the genus level, the most common genera in all groups mainly contained *Ruminococcaceae_UCG-005*, *Ruminococcaceae*_UCG-010, *Fibrobacter*,*Christensenellaceae_R-7*_*group*, *Rikenellaceae_RC9_gut_group*, and *Alistipes*; of which, the first four belonged to Firmicutes and the last two genera belonged to Bacteroidetes.

**FIGURE 2:**
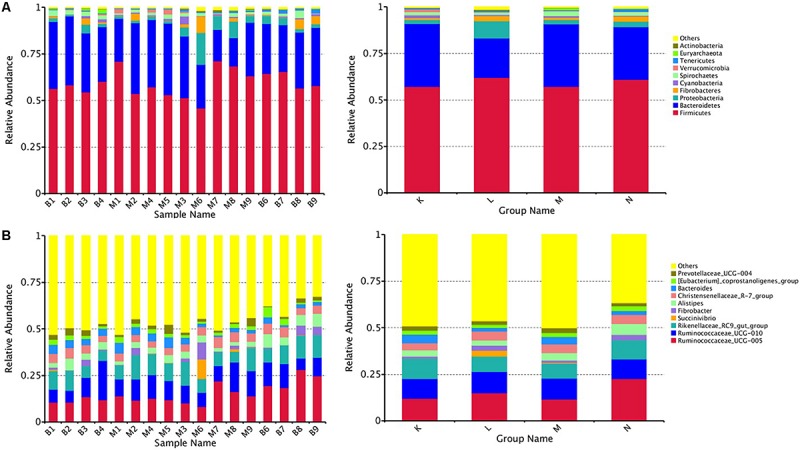
Fecal microbial composition of European mouflon and blue sheep at the phylum **(A)** and genus **(B)** levels. K, low-altitude mouflon; L, high-altitude mouflon; M, low-altitude blue sheep; N, high-altitude blue sheep.

We chose the top ten phyla and genera based on species abundance to generate a histogram, which showed the percentages of relative abundance in each sample or group (Figures 2A,B).

**FIGURE 3:**
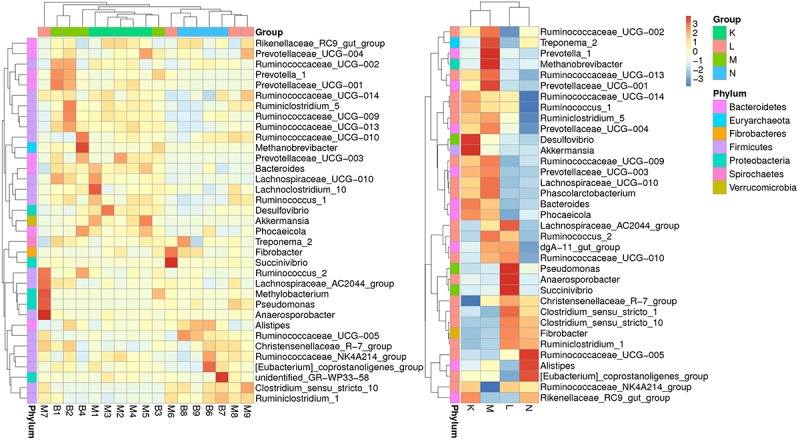
A heatmap of species abundance clustering. The heatmap shows the hierarchical clustering of samples based on the relative abundance of the top ranked 35 genera of fecal microbiota in of European mouflon and blue sheep. The relative values in the heatmap (after normalization), depicted by colors, indicate the aggregation degree or content of bacterial species among samples at the genus level. The relative values were normalized in four individual groups and displayed in figure on the right-hand side. K, low-altitude mouflon; L, high-altitude mouflon; M, low-altitude blue sheep; N, high-altitude blue sheep.

According to the relative abundance of genera in the bacterial community compositions, we selected the top ranked 35 genera to generate a clustering heatmap (Figure 3) based on relative abundance values. The values are shown in the heatmap grid with increasing abundance from blue to red. The fecal samples from mouflon and blue sheep at low altitudes (groups K and M) were grouped together while those from high altitudes (groups L and N) were clustered into another clade.

**FIGURE 4:**
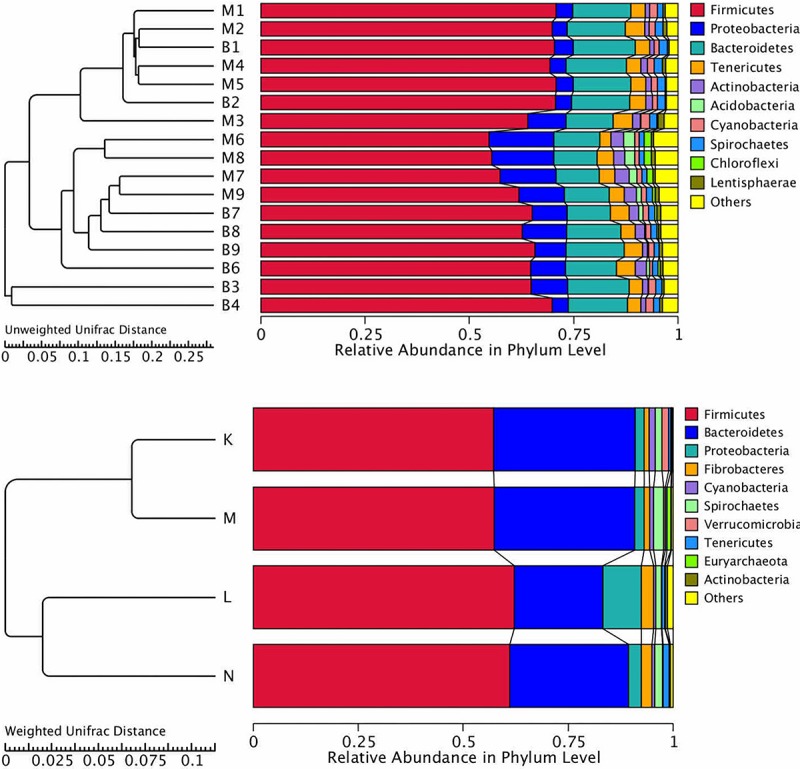
Cluster analysis with the Unifracdistance matrix. The top figure is based on the unweighted Unifrac distance and the clustering in groups. The bottom figure is based on the weighted Unifrac distance. K, low-altitude mouflon; L, high-altitude mouflon; M, low-altitude blue sheep; N, high-altitude blue sheep.

Cluster analysis using the unweighted Unifrac distance matrix was conducted following the UPGMA clustering method (Figure 4). The results were basically consistent with those from the clustering heatmap analysis.

### Analysis of Discrepancies for Between Groups

**FIGURE 5:**
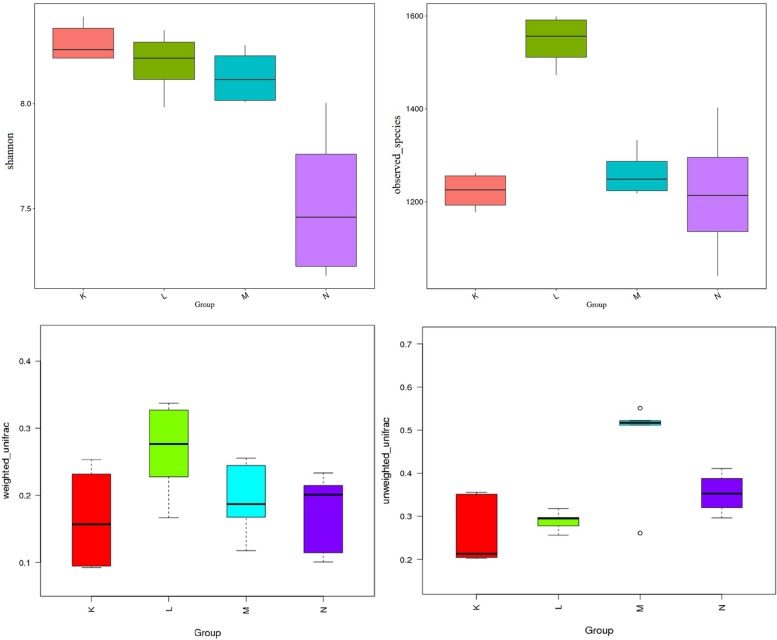
Comparisons for alpha-diversity (Shannon and observed species index) and beta-diversity (with weighted and unweighted Unifrac distance matrix) of fecal the microbiota of between the low- and high-altitude groups of sheep. K, low-altitude mouflon; L, high-altitude mouflon; M, low-altitude blue sheep; N, high-altitude blue sheep.

Turkey and Wilcoxon’s tests were used to calculate the alpha diversity (Shannon and observed species) and beta diversity in the two paired groups (Figure 5). Analysis of similarity (ANOSIM) (Table 3) and Multi-Response Permutation Procedures (MRPP) (Table 4) indicated significant differences in the bacterial communities between fecal samples collected from low and high altitudes. Notably, the differences between the samples collected from the different altitudes were considered highly significant (*P* < 0.01) in mouflon sheep and statistically significant (*P* < 0.05) in blue sheep. However, the most interesting finding was that the gut microbiota did not differ significantly (*P* > 0.05) between the mouflon and blue sheep at the same altitudes.

**TABLE 3:** ANOSIM analysis between the four groups of European mouflon and blue sheep.

**Group**	***R*-value**	***P*-value**
K-L	0.825	0.008
M-N	0.865	0.034
K-M	0.175	0.056
L-N	0.302	0.076

*K, low-altitude mouflon; L, high-altitude mouflon; M, low-altitude blue sheep; N, high-altitude blue sheep.*

**TABLE 4:** Multi-Response Permutation Procedures difference analysis between the four groups of European mouflon and blue sheep.

**Groups**	**A**	**Observed-delta**	**Expected-delta**	**Significance**
K-L	0.1450	0.3655	0.4275	0.004
M-N	0.1595	0.3800	0.4521	0.023
K-M	0.0215	0.3642	0.3722	0.051
L-N	0.0620	0.3814	0.4067	0.060

*K, low-altitude mouflon; L, high-altitude mouflon; M, low-altitude blue sheep; N, high-altitude blue sheep.*

**FIGURE 6:**
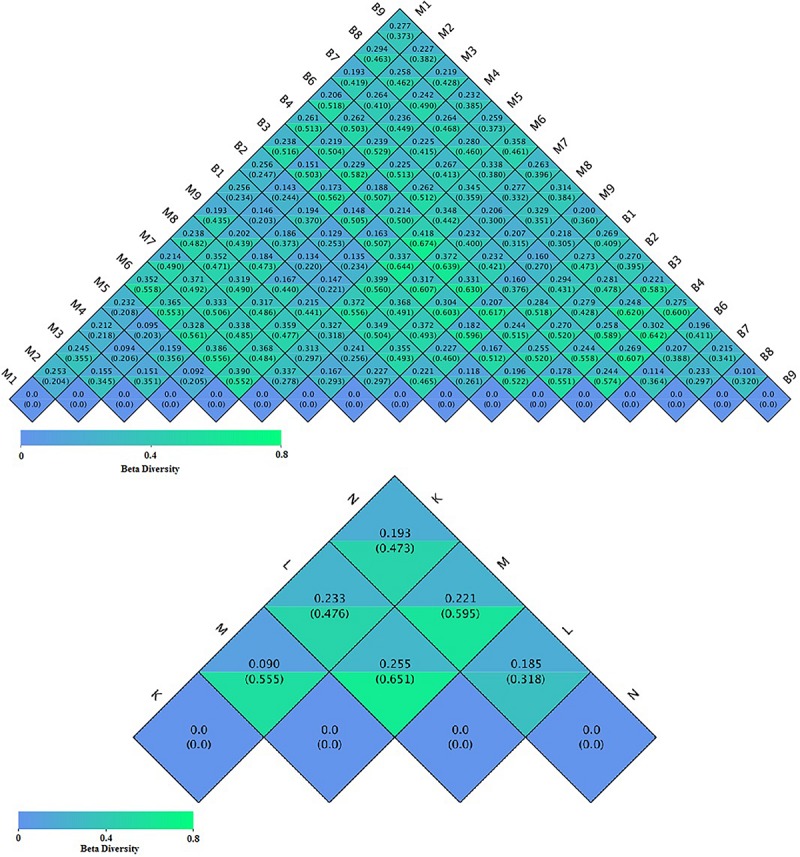
Heatmap of the beta-diversity of microbiota. The numbers in the grid blocks represent the dissimilarity coefficients between fecal samples. The top number in each block represents the weighted Unifrac distance and the bottom number represents the unweighted Unifrac distance. K, low-altitude mouflon; L, high-altitude mouflon; M, low-altitude blue sheep; N, high-altitude blue sheep.

**FIGURE 7:**
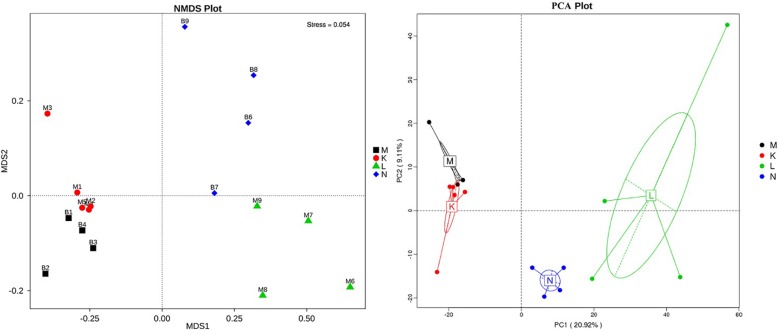
Non-metric multidimensional scaling (NMDS) and principal component analysis (PCA) of bacterial community structures. Each symbol represents the gut microbiota of an individual and the different colors represent the different groups. K, low-altitude mouflon; L, high-altitude mouflon; M, low-altitude blue sheep; N, high-altitude blue sheep.

To compare the similarity between samples and groups, a heatmap of the beta-diversity index (Figure 6) was constructed to demonstrate the correlativity based on the dissimilarity coefficient. An NMDS ordination plot (Figure 7) of bacterial taxonomy data that demonstrated the microbial differences in samples and the distance between samples reflected the degree of the discrepancy. In addition, PCAs were used to clarify the similarity of the bacterial communities in the samples collected from the same altitude (Figure 7). The gut microbiota in the mouflon and blue sheep at different altitudes were significantly different, and the bacterial population structures in the samples from the different species at the same altitudes were similar.

Additionally, we employed a *T*-test and LDA LEfSe to determine significantly different taxa between groups from different altitudes. We calculated the statistical significance using the relative abundance of the main (top 10 ranked) bacterial phyla in the mouflon (Table 5) and blue sheep (Table 6).

**TABLE 5:** Relative abundance of the top 10 most abundant bacterial phyla in the European mouflon sheep.

**Phylum**	**Avg (K)**	**SD (K)**	**Avg (L)**	**SD (L)**	***P*-value**
Firmicutes	0.572	0.080	0.621	0.113	0.497
Bacteroidetes	0.337	0.064	0.211	0.062	0.022
Proteobacteria	0.021	0.016	0.092	0.058	0.088
Fibrobacteres	0.012	0.016	0.029	0.041	0.472
Cyanobacteria	0.015	0.016	0.005	0.003	0.266
Spirochaetes	0.016	0.005	0.014	0.007	0.562
Verrucomicrobia	0.016	0.010	0.001	0.001	0.034
Tenericutes	0.006	0.003	0.006	0.002	0.766
Euryarchaeota	0.001	0.000	0.001	0.002	0.540
Actinobacteria	0.001	0.001	0.006	0.003	0.069

*K, low-altitude mouflon; L, high-altitude mouflon.*

**TABLE 6:** Relative abundance of the top 10 most abundant bacterial phyla in blue sheep.

**Phylum**	**Avg (M)**	**SD (M)**	**Avg (N)**	**SD (N)**	***P*-value**
Firmicutes	0.573	0.025	0.611	0.045	0.204
Bacteroidetes	0.335	0.035	0.283	0.029	0.065
Proteobacteria	0.023	0.018	0.029	0.013	0.578
Fibrobacteres	0.013	0.014	0.026	0.027	0.425
Cyanobacteria	0.009	0.007	0.006	0.004	0.516
Spirochaetes	0.024	0.005	0.019	0.015	0.556
Verrucomicrobia	0.004	0.003	0.001	0.000	0.148
Tenericutes	0.005	0.001	0.016	0.007	0.050
Euryarchaeota	0.009	0.008	0.001	0.001	0.136
Actinobacteria	0.001	0.001	0.002	0.001	0.518

*M, low-altitude blue sheep; N, high-altitude blue sheep.*

Significant differences were observed at the phylum level between groups K and L of mouflon sheep. The relative abundances of Bacteroidetes and Verrucomicrobia were significantly higher in the low-altitude samples (*P* = 0.021 and 0.033), and those of Acidobacteria and Chloroflexi were significantly higher in the high-altitude samples (*P* = 0.042 and 0.041). At the genus level, there were 61 genera that differed significantly between groups K and L, of which 22 of were significantly higher in the low-altitude samples while the others were significantly higher in the high-altitude samples.

Regarding differences in blue sheep at the phylum level, the relative abundance of WCHB1-60 was significantly (*P* = 0.018) higher in group N. In addition, 34 genera showed significant differences between groups M and N, of which 22 were significantly higher in group M and 12 were significantly higher in group N.

It is remarkable that the relative abundances of Firmicutes, Proteobacteria, and Fibrobacteres were slightly higher in the gut microbiota of samples collected from high altitudes in both studied animals. However, the relative abundances of Bacteroidetes and Spirochaetes were slightly higher in samples from low altitudes.

**FIGURE 8:**
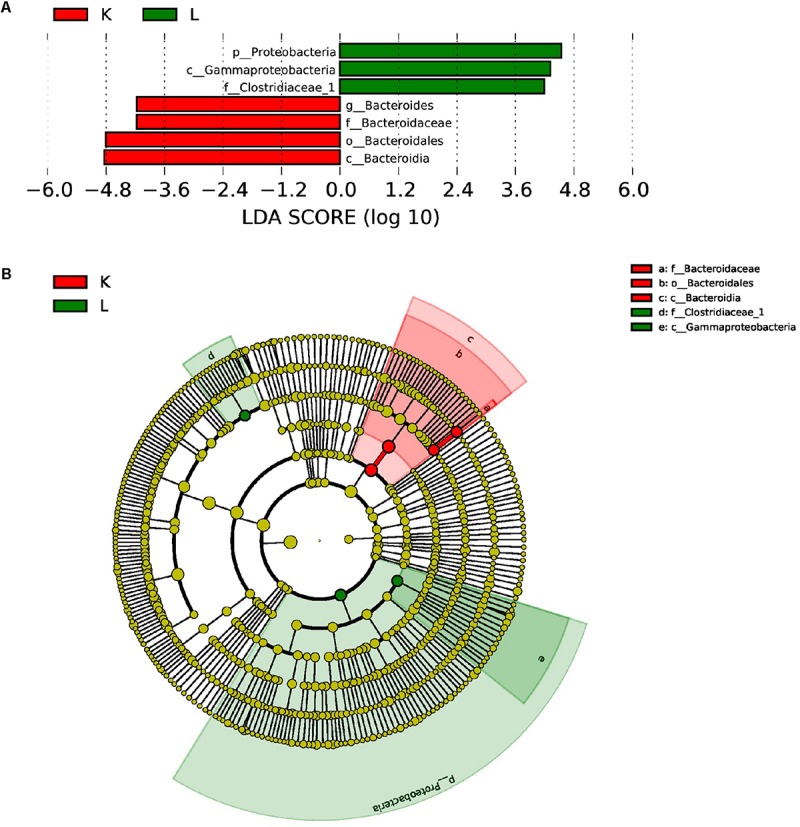
LEfSe (LDA Effect Size) analysis **(A)** and the cladogram **(B)** in European mouflon. The different colors represent the different groups. The histogram **(A)** shows the biomarkers with statistical differences between groups and the lengths of the bars indicate the influential degree of the species. The cladogram **(B)** demonstrates the classification of taxa at five levels, and the red and green circles indicate the differences in relative abundance. Non-significant differences are expressed by the yellow circles. K, low-altitude mouflon; L, high-altitude mouflon.

**FIGURE 9:**
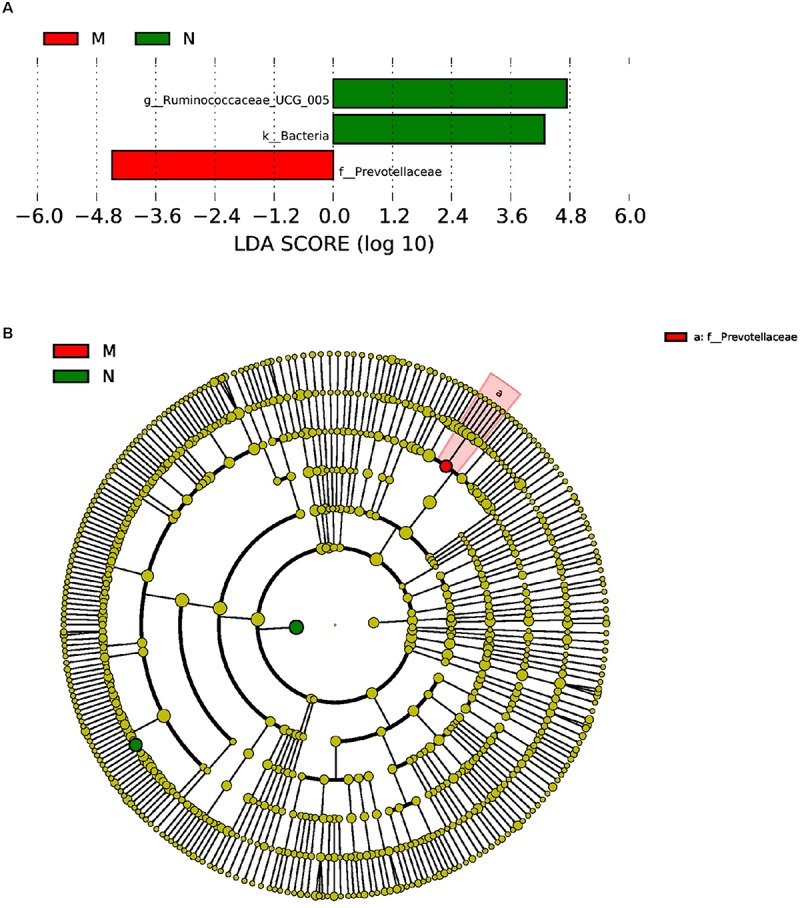
LEfSe (LDA Effect Size) analysis **(A)** and the cladogram **(B)** in blue sheep. The different colors represent different groups. The histogram **(A)** shows the biomarkers with statistical difference between groups and the lengths of the bars indicate the influential degree of species. The cladogram **(B)** demonstrates the classification of taxa at five levels, and the red and green circles indicate the differences in relative abundance. Non-significant differences are expressed by the yellow circles. M, low-altitude blue sheep; N, high-altitude blue sheep.

Furthermore, LEfSe used LDA to estimate the magnitude of the effect of altitude and provide a list of the species that showed significant differences. Seven (between group K and group L) and three (between group M and group N) biomarkers were significantly different in terms of their relative abundance in the mouflon (Figure 8A) and blue sheep (Figure 9A). Circular cladograms show the main taxa that were significantly different in the mouflon (Figure 8B) and blue sheep (Figure 9B).

## Discussion

Considering the importance of gut microbiota, the identification of factors that affected the gut microbial flora of animals may have broad implications for various fields of science, including microbiology, ecology, veterinary science, and others. Mouflon and blue sheep are considered to be primarily grazers, and in the present study we compared the gut microbial community of these two species in two independent altitudinal transects. Organisms face physiological challenges at high altitudes, such as decreases in barometric pressure, oxygen content, and temperatures. These challenges and the variations in responses to them provided unique opportunities to investigate their effects, e.g., different altitudes, on microbial composition and structure. Our results indicated that altitudinal variation was a factor that shaped the composition and structure of gut microbiota in both studied herbivores. Our findings were consistent with those of previous studies that demonstrated that altitudinal variation can affect gut microbial communities in mammals (Li H. et al., 2016; Sun et al., 2016; Zhang et al., 2016; Lan et al., 2017).

The cold and hypobaric hypoxic conditions in high-altitude habitats serve as physiologic stressors that can affect both the survival and reproduction of animals (Hochachka et al., 1996; Cheviron et al., 2012). Such stressors can cause an array of physiological changes in animals (Storz et al., 2010; Ivy and Scott, 2015; Simonson, 2015). For instance, exposure to hypobaric hypoxia can lead to increased inflammation and incidences of illness (Hartmann et al., 2000; Khanna et al., 2018). Convergent patterns have also been observed in multiple of species at high altitudes, which were consistent with the hypoxia treatment conducted in previous laboratory experiments (Suzuki et al., 2018). Moreover, weight loss was common in animals based on long sojourn times at high altitudes (Hamad and Travis, 2006; Westerterp and Kayser, 2006). The overall alpha-diversity and beta-diversity measurements, based on the rarefied OTUs in this study, were higher in the low-altitude groups than in the high-altitude groups, and a higher diversity is generally considered to be a mark of a healthy and resilient microbiota (Petersen and Round, 2014).

The Firmicutes and Bacteroidetes were the prominent phyla in both the mouflon and blue sheep, which is consistent with the findings of other studies regarding mammals (Duncan et al., 2008; Ley et al., 2008; Kong et al., 2014). Our results indicated that the groups at higher elevations had a higher relative abundance of Firmicutes and a lower relative abundance of Bacteroidetes than those at low elevations. However, the relative abundance of Firmicutes and Bacteroidetes of the gut microbiota between the mouflon and blue sheep were similar at the same altitude. Based on the understanding that microbes belonging to phylum Firmicutes contain genes related to energy metabolism and the decomposition of substances, such as fiber and cellulose (Kaakoush, 2015), and because the main functions of microbes that belong to Bacteroidetes are to degrade proteins and carbohydrates (Fernando et al., 2010; Waite and Taylor, 2014), our results suggested that the gut microbiota of high-altitude groups of sheep had stronger abilities related to energy metabolism and the decomposition of substances, e.g., fiber and cellulose, and that such abilities are associated with high-altitude adaptation. In addition, we also found that the groups at high altitudes had an increased Firmicutes:Bacteroidetes ratio for both of the two species. The high ratio of these two phyla is beneficial to animals via a gut microbiota-mediated energy harvest (Li H. et al., 2016; Li K. et al., 2016; Zhang et al., 2016; Lan et al., 2017) that can assist the host to maintain a metabolic balance and core body temperature during environmental temperature challenges (Ley et al., 2006; Turnbaugh et al., 2006; Fernando et al., 2010; Murphy et al., 2010).

At the genus level, *Christensenellaceae_R-7_group*, *Bacteroides*, *Ruminococcaceae_UCG-013*, *Prevotellaceae_UCG-003*, and *Akkermansia* were identified as the genera that differed significantly between the low- and high-altitude groups of mouflon sheep, while *Ruminococcaceae_UCG-005*, *Bacteroides*, *Ruminococcaceae_UCG-013*, *Prevotellaceae_UCG-003*, and *Prevotella_1* were the genera that showed predominant significant differences in the gut microbial flora of blue sheep. *Bacteroides*, *Ruminococcaceae_UCG-013*, and *Prevotellaceae_UCG-003* were identified in the guts of both the study species and their relative abundances were found to be higher in animals from low altitudes.

Regarding the mouflon sheep, the relative abundance of the genus *Christensenellaceae_R-7_group* (phyla: Firmicutes) was greater in the high-altitude group (*P* ≤ 0.05). Previous studies in mice, humans, and Tibetan macaques showed that the high abundance of this genus was related to a lean body shape, and some species belonging to family *Christensenellaceae* can help reduce weight gain (Goodrich et al., 2014; Oki et al., 2016; Zhao et al., 2018). Genus *Akkermansia* was the most negatively correlated bacterial genus with altitude and was an aerotolerant mucin-degrader that colonizes the mucus layer (Reunanen et al., 2015; Ouwerkerk et al., 2016). In addition, *Ruminococcaceae_UCG-005*, which is related to cellulose and starch digestion, was the predominant genus in blue sheep and the relative abundance the genus was significant higher in animals at high altitudes than in low altitudes (*P* < 0.01) (Jindou et al., 2006; Ze et al., 2012). The result suggested that *Ruminococcaceae_UCG-005* played an important role in cellulose-degradation in blue sheep at high altitudes. Interestingly, the relative abundance of *Ruminococcaceae_UCG-013* was significant higher in both studied animals at low altitudes.

*Bacteroides* are the most frequently isolated microbes from human infections, and they can cause endogenous infections in the host (Veeranagouda et al., 2014). *Prevotella*, a genus within the family Prevotellaceae, is a saccharolytic anaerobe that is associated with the consumption of a diet rich in carbohydrates and with the production of short-chain fatty acids (SCFAs) (De Filippo et al., 2010; Wu et al., 2011; Ramakrishna, 2013). *Prevotella* are known SCFA-producers, and SCFAs, such as acetic acid, can provide energy for the host and mediate blood pressure by acting on SCFA-receptors (Tremaroli and Bäckhed, 2012; den Besten et al., 2013). Moreover, a higher relative abundance of *Prevotella* can aid in the degradation of plant fibers and decrease the lipolytic and proteolytic fermentation potential (Vieira-Silva et al., 2016). The relative abundance of *Prevotella* increased dramatically in the mouflon at high altitudes, and a similar pattern was observed in pika (Li H. et al., 2016), some ruminants (Zhang et al., 2016), and humans (Li and Zhao, 2015; Lan et al., 2017). Interestingly, the relative abundance of *Prevotella* between groups of blue sheep at different altitudes was similar. However, previous studies have demonstrated negative correlations between *Prevotella* abundance and altitude (Li and Zhao, 2015; Zhao et al., 2018). We speculated that the low relative abundance of *Prevotella* in the high-altitude group may have been correlated with the short migration duration from the low to high altitudes.

Fecal microbial communities were clustered into enterotypes, determined primarily by levels of *Bacteroides* and *Prevotella* (Wu et al., 2011). The *Bacteroides:Prevotella* ratio, which is known as a dietary and lifestyle biomarker, is correlated with long-term habitual diets and provides a useful intervention target for improving host responses to environmental challenges (Gorvitovskaia et al., 2016; Costea et al., 2018). A lower *Bacteroides:Prevotella* ratio indicates a healthy, high-fiber, plant-rich diet (Gorvitovskaia et al., 2016). Previous studies revealed that a higher *Bacteroides:Prevotella* ratio is likely to indicate a high fat, high protein, and low fiber diet in animals (Wu et al., 2011). Our results indicated that the *Bacteroides:Prevotella* ratio in mouflon sheep was significantly higher in the low-altitude group (12.76) than in the high-altitude group (5.28), but in startling contrast, the *Bacteroides:Prevotella* ratio was significant higher in the high-altitude group (16.03) than in the low-altitude group (2.07) of blue sheep. The results suggested that the mouflon in the high-altitude areas were more effective in digesting high-fiber, plant-rich diets than the mouflon living at low altitudes. The differences in the *Bacteroides:Prevotella* ratio between the mouflon and blue sheep suggest that these animals may have slight differences in their nutrient digestion and metabolic mechanisms.

Moreover, there were significant differences in the relative abundance of Proteobacteria between the two altitudinal groups of mouflon sheep (*P* < 0.05). In addition, the relative abundance of Proteobacteria in blue sheep was slightly higher in the high-altitude group than in the low-altitude group, although the difference was not significant (*P* = 0.568). Proteobacteria was found to be the most predominant phylum in the giant panda and was related to lignin digestion as well as the catabolizing of various components (Evans et al., 2011; Fang et al., 2012). Due to their similar dietary composition to that of sheep, i.e., includes fiber and cellulose and other substances that are not easily digested, we inferred that the higher relative abundance of Proteobacteria observed in the present study would assist the host animals with effectively degrading their food, to obtain higher amounts of nutrients and energy.

Because altitude is an important factor that can affect and shape the gut microbial community, we attempted to understand how altitudinal variations affect the gut microbial flora in mouflon and blue sheep in the present study. Animals in high-altitude areas are exposed to hypobaric, hypoxic, and hypothermic conditions; they show increased physical activity, and even face severe dietary challenges. In our study, we collected fecal samples from study animals (under controlled dietary protocols) using non-invasive methods. Structure and cluster analyses of gut microbiota revealed that groups in the higher altitudes had a higher relative abundance of Firmicutes and a lower relative abundance of Bacteroidetes than those at low altitudes. The high Firmicutes:Bacteroidetes ratio observed in the present study is presumably beneficial to the sheep by producing gut microbiota-mediated energy. The abundance of Proteobacteria was significant higher in the gut microbiota from mouflon sheep living at high altitudes. Compared to the high-altitude groups, the *Bacteroides:Prevotella* ratio in mouflon sheep was significant higher in the low-altitude group and the ratio in blue sheep was slightly higher in the low-altitude group. The differences between the high and low-altitude groups indicated that high-altitude groups had stronger abilities related to energy metabolism and the decomposition of substances, such as fiber and cellulose. However, our understanding of the importance of altitude for the gut microbial communities of animals was limited because samples were only collected from captive animals. Furthermore, there may potentially be bacterial strains that are not identifiable using the routine procedures of next-generation sequencing technology. Thus, further investigations are needed to comprehensively understand the composition, structure, and function of gut microbial flora of European mouflon and blue sheep.

## Author Contributions

HZ, QW, GL, CZ, and WS designed the study. GS, XY, TX, and LZ conducted the research. GS, XW, YG, and YL analyzed the data. GS and QW prepared the manuscript.

## Conflict of Interest Statement

The authors declare that the research was conducted in the absence of any commercial or financial relationships that could be construed as a potential conflict of interest.
